# Targeting Wnt/Beta-Catenin Signaling in HPV-Positive Head and Neck Squamous Cell Carcinoma

**DOI:** 10.3390/ph15030378

**Published:** 2022-03-20

**Authors:** Faris F. Brkic, Stefan Stoiber, Tobias Maier, Elisabeth Gurnhofer, Lukas Kenner, Gregor Heiduschka, Lorenz Kadletz-Wanke

**Affiliations:** 1 Department of Otorhinolaryngology and Head and Neck Surgery, Medical University of Vienna, 1090 Vienna, Austria; faris.brkic@meduniwien.ac.at (F.F.B.); tobias.maier@meduniwien.ac.at (T.M.); gregor.heiduschka@meduniwien.ac.at (G.H.); lorenz.kadletz-wanke@meduniwien.ac.at (L.K.-W.); 2 Department of Pathology, Medical University of Vienna, 1090 Vienna, Austria; stefan.stoiber@meduniwien.ac.at (S.S.); elisabeth.gurnhofer@meduniwien.ac.at (E.G.); 3 Christian Doppler Laboratory for Applied Metabolomics, 1090 Vienna, Austria; 4 Unit of Laboratory Animal Pathology, University of Veterinary Medicine, 1210 Vienna, Austria; 5 CBmed GmbH—Center for Biomarker Research in Medicine, 8010 Graz, Austria

**Keywords:** Wnt, Beta-Catenin, HPV, head and neck cancer, cell culture, immunohistochemistry

## Abstract

Wnt/Beta-Catenin signaling is involved in the carcinogenesis of different solid malignant tumors. The interaction of Creb-binding protein (CBP) with Beta-Catenin is a pivotal component of the Wnt/Beta-Catenin signaling pathway. The first aim of this study was to evaluate the association of CBP expression with survival in patients with human papillomavirus (HPV)-positive head and neck squamous cell carcinoma (HNSCC). Second, the in vitro effects of the inhibition of CBP/Beta-Catenin interaction were analyzed. In particular, the effects of ICG-001, an inhibitor of CBP/Beta-Catenin interaction, on proliferation, cell death, modulation of Wnt/Beta-Catenin target expression, and cell migration were examined in vitro. High CBP expression is significantly associated with better survival on mRNA and protein levels. Furthermore, we observed cytotoxic as well as anti-migratory effects of ICG-001. These effects were particularly more potent in the HPV-positive than in the -negative cell line. Mechanistically, ICG-001 treatment induced apoptosis and led to a downregulation of CBP, c-MYC, and Cyclin D1 in HPV-positive cells, indicating inhibition of Wnt/Beta-Catenin signaling. In conclusion, high CBP expression is observed in HPV-positive HNSCC patients with a good prognosis, and ICG-001 showed a promising antineoplastic potential, particularly in HPV-positive HNSCC cells. Therefore, ICG-001 may potentially become an essential component of treatment de-escalation regimens for HPV-positive HNSCC. Further studies are warranted for additional assessment of the mechanistic background of our in vitro findings.

## 1. Introduction

Head and neck squamous cell carcinoma (HNSCC) derives from the mucosa of the larynx, oropharynx, hypopharynx, nasal cavity, and paranasal sinuses and nasopharynx. They account for almost half a million new cases annually and represent the sixth most common malignancy worldwide [1]. HNSCC is most commonly induced by carcinogens to which the mucosa of the upper aerodigestive tract is constantly exposed. Usually, exposure is a result of alcohol consumption and tobacco smoking. However, the numbers of users for these lifestyle habits are stable or slowly declining from a worldwide perspective [1,2]. In contrast, a rise in the incidence of oropharyngeal squamous cell carcinoma (OPSCC) has been observed, and the most probable underlying cause is the human papillomavirus (HPV) [2].

The role of double-stranded HPV in HNSCC, and OPSCC in particular, has been well known for the last two decades [3]. HPV is able to infect squamous cells of the upper aerodigestive tract. In particular, the oropharynx is most commonly affected. The crypts, which are widely spread in the epithelium of the tongue base and tonsils, facilitate the replication of HPV [4]. This potentially explains why HPV is found about five to ten times more often in malignancies of the oropharynx than in other head and neck areas [5].

In general, HPV-positive OPSCC is linked to excellent survival outcomes. Nevertheless, therapy for HPV-positive OPSCC is still very intense. To date, cisplatin is the most extensively used systemic agent in first-line therapeutic approaches for this type of cancer. Although cisplatin is very effective as systemic therapy, it is associated with multiple severe side effects which include, among others, acute kidney failure, hearing loss, and neuropathy [6].

In HPV-positive OPSCC, treatment de-escalation options are currently heavily discussed [7]. However, little focus lies on the identification of new tools to predict prognosis in those patients and new substances that show fewer side effects than cisplatin with similar survival outcomes. Interestingly, classical risk-factors and clinical outcome prognosticators in non-HPV HNSCC do not necessarily have the same prognostic value in HPV-positive malignancies [8]. Furthermore, a specific patient subset has a bad prognosis and/or develops distant metastases [2,9]. Finding markers that help to identify high-risk patients early might contribute to better patient care. Moreover, particularly those patients might benefit from new therapeutic targets in order to improve survival and quality of life. Currently, there are no available systemic treatment alternatives to cisplatin. For example, trials focusing on the usage of cetuximab as de-escalation treatment were inferior regarding the outcome in comparison to cisplatin [6]. Therefore, identification of novel substances that may reduce side effects while enhancing radiotherapy efficacy is warranted.

The Wnt/Beta-Catenin (WBC) pathway is an important, well-conserved signaling pathway in cancer. It is responsible for different processes during embryonic development, such as proliferation, migration, and differentiation [10]. Furthermore, it is already established that deregulation/activation of the WBC pathway can contribute to the carcinogenesis of different frequently diagnosed solid tumors, such as colorectal and ovarian cancer [11]. Recent studies indicated that activation of the canonical WBC pathway might contribute to disease progression in HNSCC [12]. However, the effects of deregulated WBC signaling in HPV-positive head and neck tumors are still unknown.

Bello et al. [13] hypothesized and reviewed the interaction between HPV infection and dysregulation of the WBC pathway. E6 and E7, two oncogenic HPV-associated proteins, significantly contribute to the malignant transformation of squamous epithelial cells. They inhibit apoptosis and induce suprabasal cell proliferation, but in addition, they may even play an important role in the deregulation of important core cancer pathways, such as the WBC pathway [14].

The small molecule inhibitor “ICG-001” blocks the interaction between Beta-Catenin and the Creb-binding protein (CBP), therefore inhibiting the WBC pathway [15]. Chemically, ICG-001 is a nitrogen-heterocyclic derivative of decahydronaphthalene with a molecular weight of 548.6 g/mol and has the molecular formula C_33_H_32_N_4_O_4_ [16]. Specifically, ICG-001 interacts with CBP and therefore competes with Beta-Catenin for CBP. Consequently, this leads to the inhibition of Beta-Catenin/CBP interaction and blocks the activation of the WBC signaling pathway, ultimately leading to a reduction of WBC-induced tumor-initiation and -proliferation [17]. Several studies have already assessed the anti-neoplastic effects and potential therapeutic value of ICG-001 for different malignant tumors, including oral [15], nasopharyngeal [18], gastric [19] and, pancreatic cancer [20].

However, to the best of our knowledge, no studies have analyzed its effects in HPV-positive malignancies yet. Therefore, our study aimed to assess the association of WBC signaling with survival in HPV-positive HNSCC. In particular, we aimed to assess the prognostic potential of CBP expression in silico. Furthermore, using in vitro experiments, we sought to analyze the antineoplastic effects of CBP/Beta-Catenin inhibition using a small molecule inhibitor ICG-001 in an HPV-positive cell line as well as HPV-negative cell line serving as a control.

## 2. Results

### 2.1. High Expression of Crebbp at mRNA Level Associates with Better Survival

The data for a total of 41 patients with HPV-positive HNSCC were retrieved from “The Cancer Genome Atlas” (TCGA). Table 1 shows the patient characteristics for the whole cohort and stratified according to the *Crebbp* mRNA expression.

**Figure 1:**
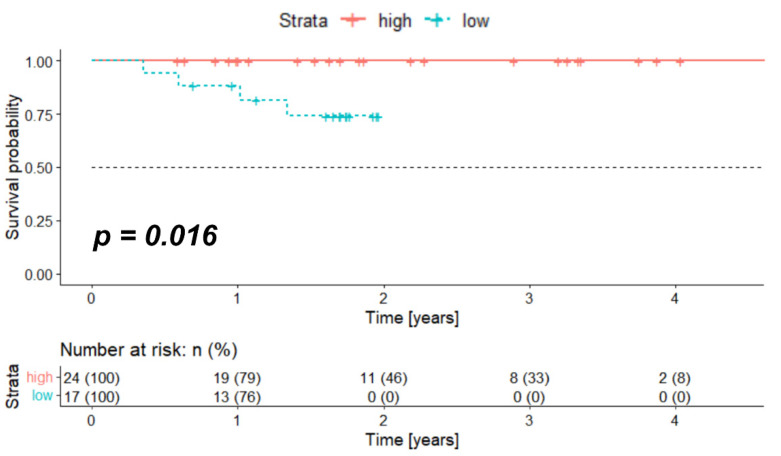
High *Crebbp* mRNA expression is associated with better survival in HPV-positive OPSCC patients. Kaplan–Meier survival curve for patients with HPV-positive OPSCC extracted from the TCGA database and stratified according to the OTV of *Crebbp*. HPV; human papillomavirus, OPSCC; oropharyngeal squamous cell carcinoma, TCGA; The Cancer Genome Atlas, *Crebbp*; CREB-binding protein, OTV; optimized threshold value.

The median overall survival (OS) for the whole cohort was 1.70 years (range 0.36–4.75 years). In order to test if *Crebbp* mRNA expression is associated with OS, the log-rank test was performed and showed significantly longer OS in the high expression group (median OS for both groups not reached due to a small number of events, *p* = 0.016). Figure 1 shows the Kaplan–Meier survival curve for the TCGA cohort stratified according to the optimized threshold value (OTV) of the *Crebbp* mRNA expression.

### 2.2. Association of CBP Protein Expression with Survival in the In-House Cohort

A total of 29 patients fulfilled the inclusion criteria and had available follow-up data. The detailed patient and tumor characteristics are presented in Table 2. Thirteen patients were female (44.8%). The median age of the whole cohort was 63.7 years (range 37.0–80.5 years). A smaller number of patients were presented with advanced local disease. Indeed, only five patients (17.2%) had a T3 or T4 tumor. Furthermore, the majority of patients had a regional cancer spread (N > 0 in 24 patients, 82.8%), and no distant metastases were observed during the initial work-up. All patients underwent a primary surgical resection, and 21 patients (72.4%) received postoperative radiotherapy.

**Figure 2:**
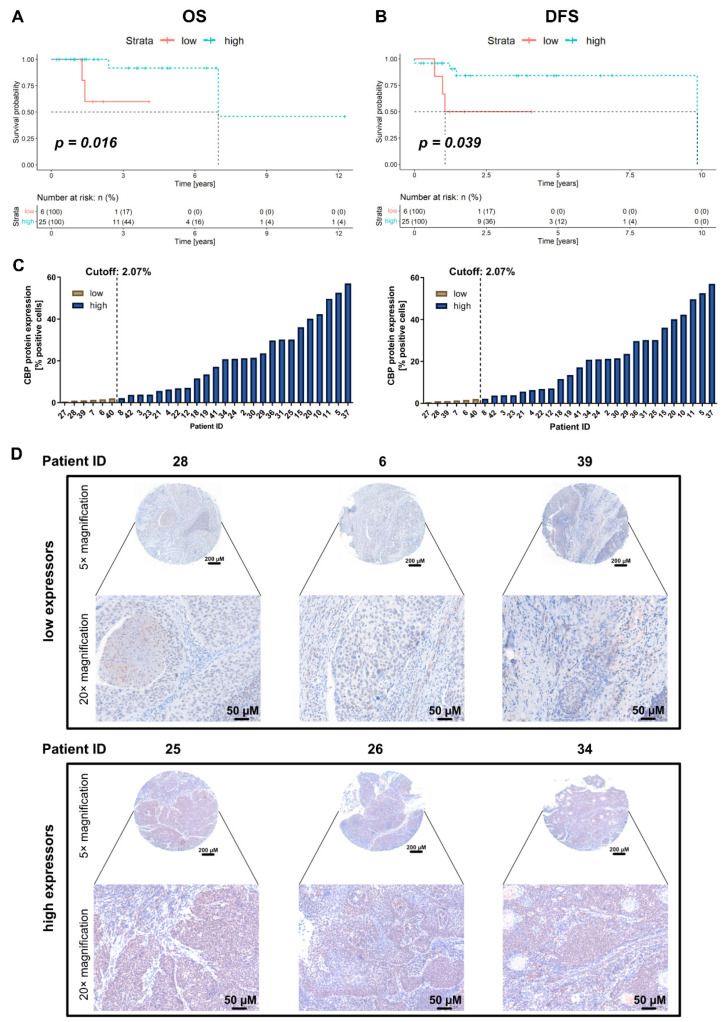
(**A**) Kaplan–Meier survival curve for OS stratified into a high and low group according to the OTV of the CBP expression. Significantly longer overall survival was observed in the group with high CBP protein expression (*p* = 0.016). (**B**) Kaplan–Meier survival curve for DFS stratified into high and low group according to the OTV of the CBP expression. Significantly longer disease-free survival was observed in the group with high CBP protein expression (*p* = 0.039). (**C**) Respective CPB expression values per patient stratified into the two groups (“low” [brown] and “high” [blue]) according to the OTV for OS (**left**) and DFS (**right**). (**D**) Representative images from the IHC staining for CBP, stratified into “low expressors” (**top**) and “high expressors” (**bottom**) according to the OTV. OS; overall survival, DFS; disease-free survival, CBP; Creb-binding protein, OTV; optimized threshold value, IHC; immunohistochemistry staining, μm; micrometer, low; the group with low CBP expression according to the OTV, high; the group with high CBP expression according to the OTV.

The median OS and disease-free survival (DFS) for the whole cohort were 1.8 years (range 0.3–12.3 years) and 1.5 years (range 0.0–9.8 years), respectively. Analog to the TCGA analysis, significantly longer survival times were observed in the group with high CBP expression as calculated by the OTV and a minimum patient distribution of 20% per group (median OS and DFS for both groups not reached due to a small number of events, *p* = 0.016 and *p* = 0.039, respectively). Figure 2A,B present the Kaplan–Meier survival curves for OS and DFS, respectively. The number of patients in each group is noted in the respective figure. In addition, Figure 2C illustrates the measured CBP expression for every patient in the two groups (low CBP expression vs. high CBP expression) based on the stratification by the OTV for OS (left) and DFS (right). Figure 2D shows images from the immunohistochemistry (IHC) staining of three independent patients for each of the two groups (low CBP expression vs. high CBP expression) with low magnification (5× magnification, total view of the tissue microarray (TMA) core) and high magnification (20× magnfication, zoomed in to the center of the core).

### 2.3. Combining ICG-001 with Irradiation Leads to a Decreased Cell Viability in a Dose-Dependent Manner in SCC154 and Cal27

**Figure 3:**
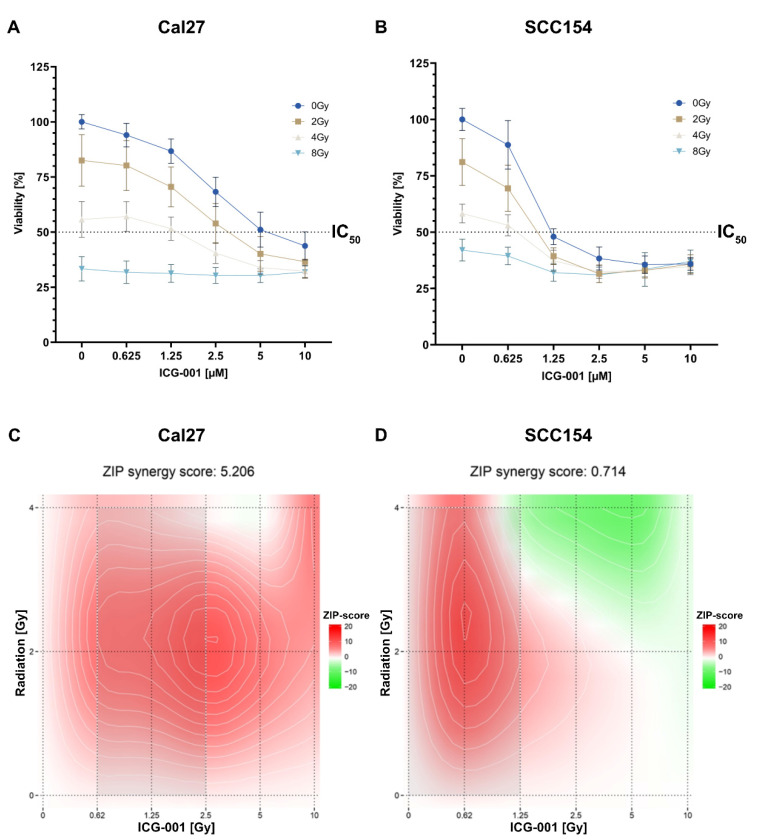
ICG-001 treatment specifically reduces cell proliferation in HPV-positive HNSCC cells in a dose-dependent manner, and its effect can be augmented by concomitant irradiation of the cells. Changes in cell viability in SCC154 (**A**) and Cal27 (**B**) cell lines following treatment with ICG-001 and irradiation. (**C**,**D**) Graphical illustration of the combinatorial analysis of ICG-001 inhibition and irradiation in regard to cell viability of Cal27 (**C**) and SCC154 (**D**) cell line. The Synergyfinder was used, and the Zip score reference model was applied. A score above 10 is regarded as synergistic interaction, between −10 and 10 as additive and less than −10 is regarded as antagonistic interaction. HPV; human papillomavirus, Gy; Gray, IC_50_; half-maximal inhibitory concentration.

Both cell lines were treated with a dilution series of ICG-001 (from 0–10 µM) in combination with irradiation (from 0–8 Grey (Gy)) in order to assess the cytotoxicity and a potential synergistic effect in combination treatment. The cell viability was measured 72 h post-treatment using a resazurin assay. A dose-dependent proliferation inhibition could be observed in both cell lines, and the IC_50_ was notably lower in the HPV-positive HNSCC cell line SCC154 than in HPV-HNSCC cell line Cal27 (0.88 µM and 4.65 µM, respectively; Figure 3A,B). The combinatorial effects of ICG-001 and irradiation are graphically illustrated in Figure 3C,D (Cal27 and SCC154, respectively). Relative mean Zip-synergy scores of 0.714 and 5.206 were calculated for SCC154 and Cal27, respectively. Overall, mostly additive effects were observed for both cell lines, and the strongest (synergistic) effects were calculated at a combination of 2 Gy irradiation and 2.5 µM of ICG-001 for Cal27 and 0.625 µM of ICG-001 for SCC154. According to calculated IC_50_ values for the two cell lines, different ICG-001 concentrations were used in subsequent experiments. In particular, SCC154 was treated with lower inhibitor concentrations than Cal27.

### 2.4. Treatment with ICG-001 Downregulates CBP and Shows Different Down-Stream Modulation of Cell-Cycle Control between HPV-Negative and HPV-Positive HNSCC Cells

**Figure 4:**
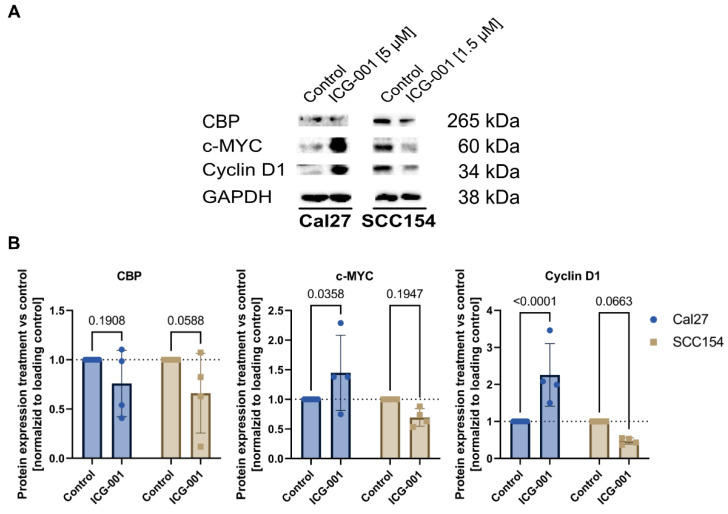
ICG-001 treatment leads to a downregulation of CBP and shows differential effects on WBC target genes in HPV-negative and HPV-positive HNSCC cells. (**A**,**B**) Representative immunoblotting results and quantification for CBP, c-MYC, and Cyclin D1 expression in control and ICG-001-treated cells. Cells were treated with ICG-001 for 72 h. Bar plots represent mean ± SD. Statistical differences between control and ICG-001-treated cells were assessed using the one-way ANOVA test. HPV; human papillomavirus, CBP; Creb-binding protein, WBC; Wnt/Beta-Catenin, SD; standard deviation.

We could generate evidence that ICG-001 treatment leads to a downregulation of CBP (Figure 4A,B). In addition, screening for two bona fide transcriptional targets of the WBC pathway revealed that ICG-001 treatment leads to a strong repression of Cyclin D1 and c-MYC (two strong drivers of cell survival and proliferation and WBC pathway target genes [10,11,12]) in the HPV-positive cell line SCC154, whereas in the HPV-negative cell line Cal27, an upregulation thereof was observed (Figure 4A,B). The increased levels of Cyclin D1 and c-MYC in the HPV-negative cell line (Cal27) post-ICG-001 treatment potentially compensate partially for the anti-neoplastic-effects of ICG-001 treatment—hence limiting the overall efficacy of ICG-001 in the HPV-negative Cal27 cell line.

### 2.5. Treatment with ICG-001 Induces Apoptosis in SCC154 and Cal27 Cells

**Figure 5:**
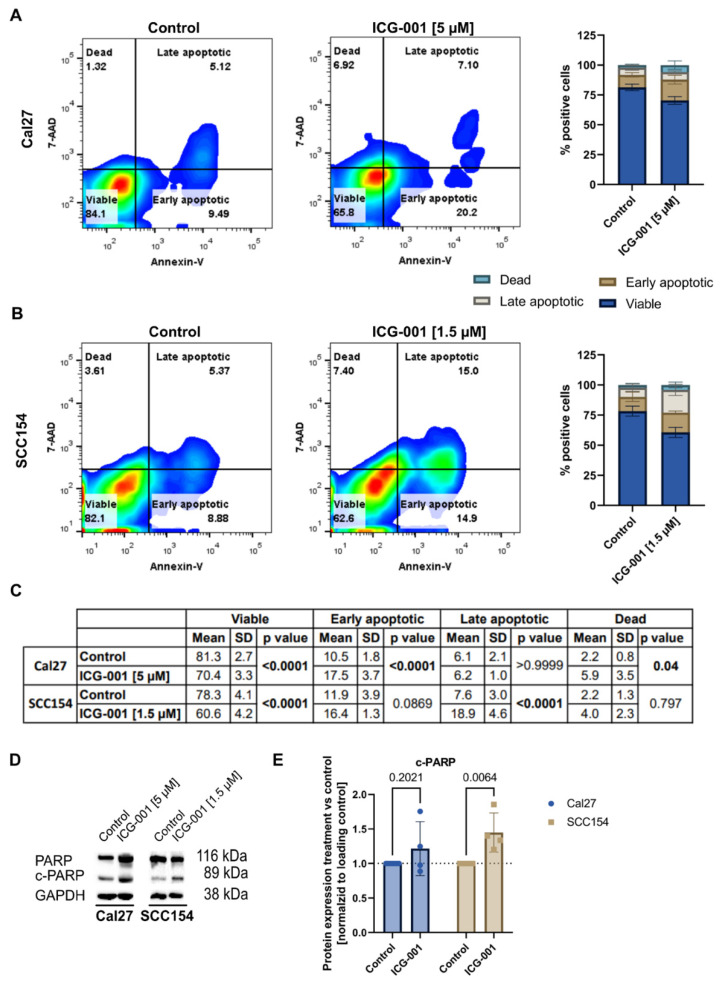
ICG-001 treatment induces apoptosis in HPV-negative and HPV-positive HNSCC cells. (**A**) Representative dot plots of Annexin V/7-AAD assay performed after 72 h of treatment with ICG-001. One plot each is shown for control (untreated) and ICG-001-treated cells for Cal27 and SCC154, respectively (**A**,**B**). Proportion of “viable”, “early apoptotic”, “late apoptotic”, and “dead” cells in control and in treated cells was quantified and is shown in the staggered bar plots as mean ± SD (**A**,**B**). Numeric representation of the quantification of the Annexin V / 7-AAD assay (**C**). Immunoblotting results and quantification for PARP and c-PARP expression in control as well as in ICG-001-treated cells after 72 h of treatment. Bar plots represent mean ± SD (**D**,**E**). Statistical differences between control and ICG-001-treated cells were assessed using the one-way ANOVA test (Immunoblotting results) and the two-way ANOVA test (Annexin V/7-AAD results). HPV; human papillomavirus, PARP; Poly(ADP-ribose)-Polymerase, SD; standard deviation.

A significant change in the amount of early apoptotic, late apoptotic, and dead cells could be observed in one or both tested cell lines post 72 h treatment with ICG-001 at 1.5 µM and 5 µM, respectively. In particular, the early apoptotic cell percentage increased significantly in Cal27 cells upon treatment (10.5 ± 1.8% vs. 17.5 ± 3.7%, Figure 5A,C). Moreover, a significant increase in dead cells could be detected in ICG-001-treated Cal27 cells (2.2 ± 0.8% vs. 5.9 ± 3.5%, Figure 5A,C). Interestingly, for the HPV-positive cell line (SCC154), the amount of late apoptotic cells significantly differed between control and ICG-001-treated cells (7.6 ± 3.0% vs. 18.9 ± 4.6%, Figure 5B,C)—potentially indicating that these cells already entered apoptosis earlier than the Cal27 cells. Furthermore, in both cell lines, the fraction of viable cells was significantly different between the control and treated sample (Cal27: 81.3 ± 2.7% vs. 70.4 ± 3.3%, SCC154: 78.3 ± 4.1% vs. 60.6 ± 4.2%, Figure 5A–C). Combined with the increase of cleaved PARP (c-PARP) in ICG-001-treated samples (Figure 5D), this strongly indicates that WBC pathway blockade via ICG-001 induces apoptosis in the tested cell lines, especially in the HPV-positive cell line SCC154.

### 2.6. Treatment with ICG-001 Shows Antimigratory Effects In Vitro

**Figure 6:**
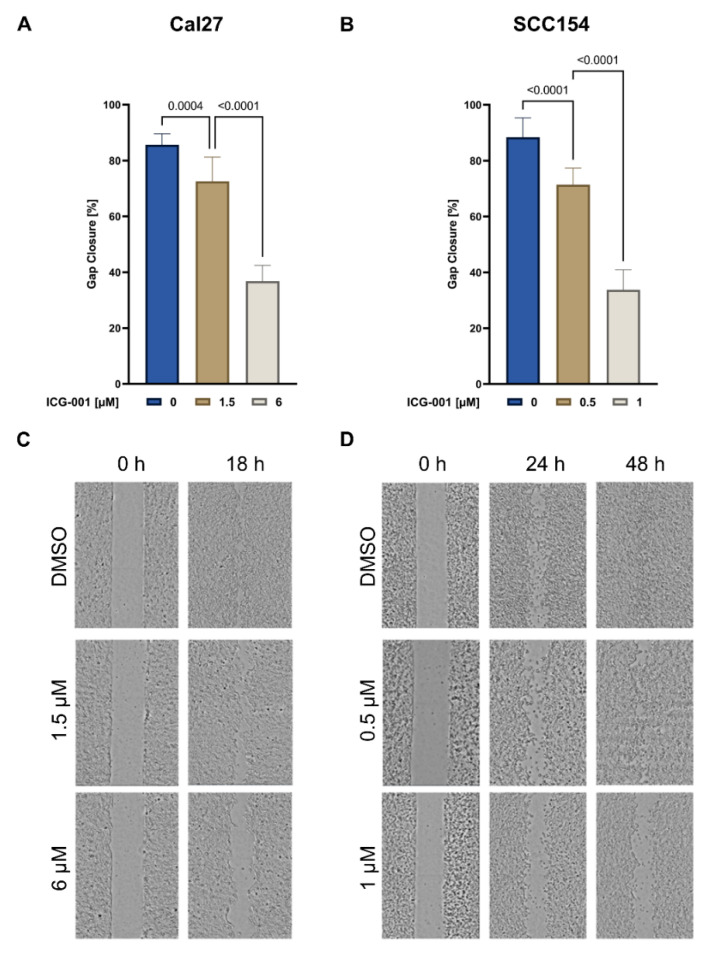
ICG-001 treatment reduces the migratory potential of HPV-negative and HPV-positive HNSCC cells. (**A**,**B**) Graphical illustration of the analyzed migration assay of Cal27 (0 and 18 h, (**A**) and SCC154 (0 and 48 h, (**B**)) cells treated with ICG-001 or vehicle control (DMSO). With lower inhibitor concentrations, a similar anti-migratory effect was observed in the SCC154. (**C**,**D**) Visualization performed with the TECAN reader for Cal27 (**C**) and for SCC154 (**D**) at the indicated time points. The free area was measured immediately after the gap generation, as well as after 24 h and 48 h in SCC154. Due to faster cell migration and gap closure, the second measurement was performed after 18 h in Cal27. Statistical differences between control and ICG-001-treated cells were assessed using the one-way ANOVA test. HPV; human papillomavirus, DMSO; dimethyl sulfoxide, SD; standard deviation.

Cal27 and SCC154 were treated with 1.5 and 6 µM, and 0.5 and 1 µM of ICG-001, respectively. The anti-migratory effects were assessed by the wound healing tool in ImageJ after 18 h in Cal27 and after 48 h in SCC154 (Figure 6A,B). Cal27 cells treated with ICG-001 showed a gap closure of 72.6% (1.5 µM, Figure 6A,C) and 36.81% (6 µM, Figure 6A,C), whereas DMSO-treated Cal27 cells showed a gap closure of 85.73% (Figure 6A,C. In SCC154 cells treated with ICG-001, the calculated gap closure measured 71.44% (0.5 µM, Figure 6B,D) and 33.76% (1 µM, Figure 6B,D) in contrast to 88.43% in the DMSO-treated control group (Figure 6B,D). Overall, ICG-001 treatment showed a significant reduction of cell migration for both cell lines; indeed, for SCC154 cells, lower concentrations were needed to obtain a similar inhibitory effect.

### 2.7. Proposed Mechanism of ICG-001 in the Tested HNSCC Cell Lines

**Figure 7:**
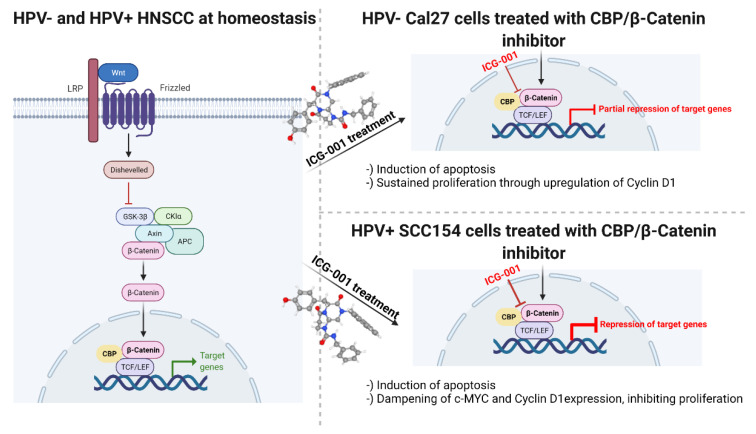
The mechanism of action of ICG-001 in HPV-negative and HPV-positive HNSCC cells. Schematic representation of WBC signaling at baseline (homeostasis) and the effects of CBP inhibition via ICG-001 (the molecular structure was retrieved from the publicly available and free reproducible source [16]) in HPV-negative and HPV-positive HNSCC tumor cells. The scheme was created with BioRender.com (accessed on 30 December 2021).

Our in vitro findings on the mechanism of action of ICG-001 in HPV-negative Cal27 and HPV-positive SCC154 cells are graphically summarized in Figure 7.

## 3. Discussion

The current study revolved around the role of the WBC pathway in HPV-positive HNSCC in terms of its prognostic relevance and therapeutic potential of its inhibition. First, we provided evidence that high *Crebbp* mRNA expression is associated with better survival in patients with HPV-associated HNSCC. These results could be validated in an independent, in-house cohort on protein level. Therefore, CBP expression can facilitate the identification of patients with a potentially better prognosis. Furthermore, in our in vitro experiments, we presented anti-migratory, proapoptotic, cytotoxic, as well as synergistic effects with radiotherapy of CBP/Beta-Catenin inhibition via ICG-001 in both cell lines, SCC154 and Cal27. Effects were indeed more potent in the HPV-positive cell line (SCC154). Moreover, downregulation of WBC transcriptional targets was observed in ICG-001-treated SCC154 cells, further supporting the hypothesis of HPV-induced deregulation of the WBC pathway.

The interaction between CBP and Beta-Catenin is an important component of the WBC pathway [21]. Therefore, we first evaluated the association of *Crebbp* mRNA and protein expression with survival in HPV-positive HNSCC in silico. High CBP expression was linked to better survival times. Similar results on protein level were shown by Rühlmann et al. [22] for patients with locally advanced rectal cancer. The mechanistic background behind the association of high CBP expression with better survival in HPV-positive HNSCC patients certainly warrants further elucidation. One possible explanation is the proposed association of higher HPV viral load with better survival in patients with an HPV-associated malignancy [23]. Higher viral load is supposed to induce a higher expression of HPV oncogenes E6 and E7. Rampias et al. [24] were able to observe an E6 and E7-mediated upregulation of Wnt/Beta-Catenin signaling. Thus, overexpression of Wnt-associated genes (including *Crebbp*) could be the result of a higher viral load and have a similar positive prognostic effect, such as the high viral load, in those tumors. Indeed, further studies are needed in order to decipher the exact mechanistic background behind this observation.

The above-noted interaction of the HPV with the WBC pathway via the HPV-associated oncoproteins E6 and E7 was further elucidated in a recent study by Muñoz-Bello et al. [25]. They were able to show that E6 with its spliced isoform upregulates the WBC signaling cascade through the transcription factor TCF-4. In particular, WBC target gene overexpression is achieved by upregulation of TCF-4 via E6 and its spliced isoform. We investigated the therapeutic potential of WBC inhibition by ICG-001 in HNSCC cell lines with a specific focus on the HPV-positive HNSCC cell line SCC154. We show here for the first time a downregulation of WBC signaling induced by CBP/Beta-Catenin inhibition via ICG-001. The treatment with ICG-001 resulted in pronounced early apoptotic and late apoptotic cell death, which was also observed in Cal27, albeit to a lesser extent than in SCC154. Treatment with ICG-001 specifically decreases cell proliferation in the HPV-positive HNSCC cell line in a dose-dependent manner, and its effect can be enhanced by simultaneous irradiation in both tested cell lines. Furthermore, our data support the observation that HPV-positive HNSCC shows a better response to radiotherapy [26] than HPV-negative tumors. Similarly, ICG-001 increased radiosensitivity in a hepatocellular carcinoma cell line [27] and reversed radiotherapy resistance in a non-small cell lung cancer cell line [28]. In addition, we show high anti-migratory effects of ICG-001 treatment on the HPV-positive cell line. Up to now, this was the first study analyzing the anti-migratory effects of ICG-001 for an HPV-positive HNSCC cell line (SCC154). Interestingly, lower inhibitor concentrations were needed to achieve a reduction of cellular migration in SCC154 compared to Cal27. Similarly, antiproliferative and proapoptotic effects were stronger in SCC154 than in Cal27. This further supports the hypothesis that HPV may interfere with the activation of the WBC pathway, and hence, stronger antineoplastic effects of CBP inhibition are observed in HPV-positive cancer cells. Hypothetically, HPV infection might lead to an overexpression of CBP by activating WBC pathway transcription via E6-regulated TCF-4. Moreover, the interaction between E6 and E7 HPV oncoproteins and the nuclear accumulation of active Beta-Catenin in HPV-positive oropharyngeal cell line was shown by Rampias et al. [24] They even proposed Beta-Catenin as a biomarker for developing an HPV-induced carcinoma.

In the study by Schultz et al. [29], the expression of the Beta-Catenin as well as of the c-kit was assessed in p16-positive, and HPV-negative HNSCC treated with imatinib. However, this study lacks in vitro analyses of the therapeutic potential of the Wnt/Beta-Catenin signaling blockade. On the other hand, we show here for the first-time mechanistic evidence that the antineoplastic effects induced by ICG-001 are specific for an HPV-positive HNSCC cell line (SCC154). ICG-001 strongly downregulated the transcriptional targets c-MYC and Cyclin D1 in the HPV-positive cell line SCC154, which was in contrast to the HPV negative cell line Cal27, where we observed an upregulation of both proteins. In our opinion, this different modulation may explain the dissimilar sensitivity of the two cell lines to ICG-001 in our experiments.

The various side-effects of cisplatin, including acute kidney failure or hearing loss [6], are well-known and impose an important limitation. Therefore, other chemotherapy options or add-on therapies for patients with HPV-positive OPSCC are certainly needed. On the other hand, small molecule inhibitors that are currently in clinical use or studies were associated with low side-effect-rates [30,31]. Furthermore, the safety of PRI-724, a small-molecule inhibitor of CBP/Beta-Catenin interaction and an analog of ICG-001, was demonstrated in phase 1 clinical trial of Kimura et al. [32]. Moreover, Lenz et al. [33] summarized preclinical and clinical studies regarding PRI-724 safety. The perhaps most interesting trial comprised 18 patients continuously receiving rising doses from 40 to 1280 mg/m^2^/day for 1 week. Indeed, the cytotoxicity rate was acceptable, with only one patient developing hyperbilirubinemia [34]. Ko et al. [35] conducted a phase Ib study on PRI-724 with gemcitabine as a second-line treatment for advanced pancreatic adenocarcinoma. Similarly, a low side-effect profile, as well as the satisfactory clinical activity of PRI-724, were recorded. Although still not elucidated, it can be hypothesized that the clinical toxicity of ICG-001 should be comparable to PRI-724. Interestingly, CBP/Beta-Catenin inhibitors are not necessarily potential treatment agents only for tumors with overexpressed canonical Wnt pathways. Indeed, Deng et al. [36] reported on the therapeutic effects of ICG-001 in *neurofibromin 2* gene mutated meningioma, using in vitro and in vivo xenograft models.

As noted, the majority of patients with HPV-positive OPSCC associate with good outcomes. Therefore, efforts are made to investigate possible treatment de-escalation regimens [7,37]. These should contribute to a reduction of treatment morbidity while maintaining comparable efficiency. As CBP was highly expressed in patients with good survival rates, the CBP/Beta-Catenin targeted chemotherapy agents, which are associated with low cytotoxicity, may potentially have future clinical utilization as a part of de-escalation regiments in HPV-positive OPSCC.

Limitations of the current study include that the size of the TCGA cohort is relatively small. Nevertheless, a significant association between high *Crebbp* expression and better survival outcome was observed in silico and validated on protein level in an independent cohort. For future validation studies of our in vitro data, additional HPV-negative and HPV-positive cell lines should be included. In addition, the current study did not provide information on the exact mechanistic effects of ICG-001 treatment in vitro, particularly for HPV-negative cells. Despite the above-mentioned shortcomings of the study, CBP expression may become a valuable survival biomarker for HPV-positive HNSCC. Moreover, the ICG-001 may become a candidate for targeted therapy in HPV-associated HNSCC. Taken together, we showed that CBP expression might be a valuable prognostic marker and the specific blockade of its interaction with Beta-Catenin a potential therapy option for low-risk patients with HPV-positive HNSCC.

## 4. Materials and Methods

### 4.1. The Cancer Genome Atlas (TCGA)—TCGA Cohort

First, the association of CBP expression on mRNA level (gene name of CBP: *Crebbp*) with OS in HPV-positive HNSCC was analyzed. The data were retrieved using the TCGA database (https://portal.gdc.cancer.gov/projects/TCGA-HNSC. accessed on 1 March 2021) [38]. All patients with a primary treated and HPV-positive HNSCC were included in the analysis. Besides all available clinical and follow-up data, gene expression results from Illumina HiSeq RNA sequencing (RNA-seq) were downloaded from the GDC Legacy Archive (https://portal.gdc.cancer.gov/legacy-archive/search/f. accessed on 30 December 2021) and normalized utilizing the R package “TCGA biolinks” (version 2.19.0) and processed in R (version 4.0.3, R Foundation for Statistical Computing, Vienna, Austria). Survival analysis was performed with packages “survival” and “survminer” (versions 3.2.13 and 0.4.9, respectively) in R. Kaplan–Meier curves were plotted with “ggplot” (version 3.3.3). The OTV of *Crebbp* expression in regard to OS was calculated (>OTV was considered as high expression) with a patient distribution of at least 20% per group. The whole cohort was stratified according to the OTV into high and low and a Kaplan–Meier survival curve for OS was calculated and plotted.

### 4.2. Immunohistochemistry—The MUV Cohort

All patients with a histologically verified HPV-positive HNSCC, primarily surgically treated in our institution from 1 January 2012 to 31 December 2019, were included in this study. Patients with recurrent disease or a second malignancy were excluded from the analysis. Furthermore, data on age, sex, tumor staging, and other clinically relevant data were retrieved. The association of CBP expression on protein level with OS and DFS was analyzed.

For this analysis, a TMA of formalin-fixed paraffin-embedded tumor samples of all included patients was constructed. This was performed with the computer-assisted tissue microarray platform (TMA Grand Master, 3D Histech, Budapest, Hungary). The subsequent staining was performed on 4 µm slices.

The immunohistochemical staining was performed with the Lab Vision UltraVision Kit (Thermo Scientific, Waltham, MA, USA, TL-060-HL), with colon tissue serving as a positive control. For the staining, the TMA was first dewaxed and dehydrated. Then, antigen retrieval was performed using ethylenediaminetetraacetic acid in a microwave (10 min (min) at 600 Watt). Next, 3% H_2_O_2_ and Ultra V Block were used in order to block the endogenous peroxidase activity. Then, the samples were incubated for 1 h (h) with the primary antibody against CBP 1:100 (Santa Cruz, sc-7300) at room temperature. Subsequently, the primary antibody enhancer and horseradish peroxidase enhancer were applied for 10 and 15 min, respectively. In order to visualize the staining, the UltraVision Plus Detection System DAB Plus Substrate System (Thermo Scientific, Waltham, MA, USA, TL-060-HL) was utilized. The counterstaining of the tissues was performed with hematoxylin Gill II (Merck, Darmstadt, Germany, 105175). The tissues were scanned using the Panoramic Flash 250 II. The expression of the CBP was analyzed. We performed the quantification of the staining using QuPath (Version 0.2.3). Tissue was analyzed for the percentage (0–100%) of stained cells. The survival analysis was conducted with the R packages “survival” and “survminer” (R: A language and environment for statistical computing. R Foundation for Statistical Computing, Vienna, Austria. URL https://www.R-project.org/, accessed on 23 October 2020). The optimized threshold values in regards to overall and DFS were calculated, serving as a cutoff for the patient stratification into high and low CBP groups (>optimized threshold was considered as high). The calculation of the optimized threshold was performed with a patient distribution of at least 20% per group. Kaplan Meier curves were plotted with “ggplot”.

### 4.3. Cell Culture

Next, we evaluated the effect of WBC pathway inhibition in vitro. For these experiments, we used the HPV-positive HNSCC cell line SCC154. The Cal27, an HPV-negative HNSCC, cell line served as a control. They were both acquired from the American Type Culture Collection (ATCC, Manassas, VA, USA) and were regularly tested for mycoplasma contamination. Dulbecco’s modified eagle’s medium (DMEM), Penicillin/Streptomycin (P/S) and fetal calf serum (FBS) were obtained from Gibco (Gibco, Thermo Fisher Scientific, Waltham, MA, USA). Cells were kept in a humidified environment at 37 °C and 5% CO_2_ (Hera Cell 240, Heraeus Holding GmbH, Hanau, Germany) and cultivated in DMEM supplemented with 10% FBS and 1% P/S. Cells were split once per week by trypsinization. The inhibitor ICG-001 was acquired from Selleckchem (Selleckchem S2662, Houston, TX, USA). All in vitro experiments were performed at least three times. Mean values ± standard deviations (SD) were calculated and used for further analysis and graphical representation. Displayed graphical presentation show representative images.

### 4.4. Cytotoxicity Assay

In order to analyze cell proliferation, cells were seeded in 96-well plates (Sarstedt, Nürnbrecht, Germany) in the following fashion: SCC154 20,000 cells/100 µL/well; Cal27 5000 cells/100 µL/well. A set of five replicates for each dose was used. One day after seeding, cells were treated with 100 µL of inhibitor diluted in DMEM. Ascending inhibitor concentrations were used and ranged between 0.625 and 10 µM. Dimethyl sulfoxide (DMSO) (Sigma-Aldrich, D8418, St. Louis, MO, USA) treated cells served as control. Furthermore, cells were irradiated at 2, 4, and 8 Gy (YXLON International GmbH, Hamburg, Germany). The Synergyfinder tool (https://synergyfinder.org, accessed on 31 January 2021) was used for calculating synergistic/additive effects of irradiation and ICG-001 treatment.

After 72 h of treatment, the medium was removed, 100 µL of 56 µM Resazurin (Sigma-Aldrich, St. Louis, MO, USA), diluted in DMEM, was added to each well, and the cells were incubated. The measurements were performed with a TECAN Spark reader (Tecan Group, Männedorf, Switzerland); for Cal27 after 90 min of incubation and for SCC154 after 150 min.

Based on IC_50_ inhibitor values calculated in the cytotoxicity assay, corresponding inhibitor concentration ranges of ICG-001 were used in subsequent experiments for two cell lines.

### 4.5. Migration Assay

In order to assess the anti-migratory effects of WBC inhibition in a dose-dependent manner, the cells were seeded in 24-well plates (Greiner Bio-One, Frickenhausen, Germany) with 150,000 Cal27 cells/well and 700,000 SCC154 cells/well. The medium was prepared with DMEM containing 1% P/S and 1% FBS (in order to starve the cells, thereby limiting their proliferation). Cells were seeded at 100% confluency, and simultaneously, a migration gap was created by a culture insert (Ibidi, Gräfelfing, Germany) which was removed 24 h after seeding. Then, the cells were washed with 1x DPBS (Gibco, Thermo Fischer Scientific, Waltham, MA, USA) once followed by treatment with ICG-001. Concentrations of 1.5 and 6 µM in Cal27 and 0.5 and 1 µM in SCC154 of ICG-001 were used.

The initial visualization was performed immediately after treatment with a TECAN Spark reader. The same was performed after 24 and 48 h in SCC154 and after 18 h in Cal27 (as the gap was already closed after this time). Before visualization, cells were washed with 1x DPBS, and fresh DMEM was added (in order to remove dead and floating cells). The analysis and calculation of the gap closure percentage were performed with ImageJ 1.53e [39].

### 4.6. Cell Apoptosis: Fluorescence-Activated Cell Sorting (FACS)—Annexin V Assay

In order to assess cell apoptosis after treatment with ICG-001, a FACS Annexin V assay was performed. Firstly, 80,000 Cal27 and 150,000 SCC154 cells were seeded into each well on a 12-well plate. On the next day, the cells were either treated with DMSO (vehicle control) or the approximate IC_50_ concentrations of ICG-001 (5 µM for Cal27 and 1.5 µM for SCC154). After 72 h of treatment, the cells were trypsinized, washed, and pelleted, and the apoptotic cell death was determined by Annexin V (Invitrogen, Thermo Fisher Scientific, R37174, Waltham, MA, USA)/7-AAD (Abcam, ab228563, Cambridge, UK) co-staining, following the instructions from the manufacturer. Data from stained cells were acquired with a BD FACSCanto II (Becton Dickinson, Franklin Lakes, NJ, USA). Analysis of results was performed with the FACSDiva software 8.0.1 (Becton Dickinson, Franklin Lakes, NJ, USA).

### 4.7. Immunoblotting

After trypsinization, washing, and pelleting of the cells, the cell pellet was lysed in RIPA buffer (Sigma-Aldrich, D8418, St. Louis, MO, USA) supplemented with phosphatase (PhosSTOP™, Roche, Basel, Switzerland) and protease inhibitors (complete protease inhibitors, Roche, Basel, Switzerland). For all samples, equivalent amounts of protein were diluted in RIPA buffer and separated by a 10% SDS-PAGE. Next, proteins were transferred to a nitrocellulose membrane (Millipore, Burlington, MA, USA) with a Trans-Blot Turbo Transfer System (Bio-Rad, Hercules, CA, USA). In the following step, membranes were blocked with 5% BSA in 1 × TBS/0.1% Tween-20 for 1 h and incubated overnight at 4 °C on a roller shaker with the following primary antibody: β-TUBULIN (1:1000, Cell Signaling Technology, #86298, Danvers, MA, USA), PARP (1:1000, Cell Signaling Technology, #9542, Danvers, MA, USA), CBP (1:500, Santa Cruz, sc-7300, Santa Cruz Biotechnology, Dallas, TX, USA), c-MYC (1:500, Cell Signaling Technology, #18583, Danvers, MA, USA) and Cyclin D1 (1:1000, Cell Signaling Technology, #55506, Danvers, MA, USA). On the following day, membranes were incubated with the second antibody (specific for the host species of the primary antibody) at a dilution of 1:5000 (in 1× TBS / 0.1% Tween-20) for 1 h at room temperature. The target protein expression was visualized using the ECL™ Prime Western Blot System from Cytiva (Merck, Darmstadt, Germany) and the ChemiDoc XRS+ (Bio-Rad, Hercules, CA, USA). Quantification of results from immunoblotting was conducted using ImageJ 1.53e [39].

### 4.8. Statistics

The statistical analysis of the patient cohort data was performed with the R software. The level of statistical significance was set at 0.05, two-tailed. Survival analysis was performed using R, as described above, and the log-rank test was used to assess if the two groups have a statistically significant difference in survival times.

In order to assess the differences in gap closure between different inhibitor concentrations in the migration assay and to analyze results from the Annexin V/7-AAD assay, the two-way ANOVA test was used. Differences in protein expression between treated and untreated cells were assessed using the one-way ANOVA test. Graphical presentation of cell culture results, as well as the one-way/two-way ANOVA analyses, were performed with GraphPad Prism (GraphPad Prism version 8.4.2 for Windows, GraphPad Software, www.graphpad.com. accessed on 5 December 2020).

## 5. Conclusions

In the current study, we provided novel insights into the potential therapeutic value of WBC inhibition in HNSCC, particularly in the HPV-positive cell line SCC154. ICG-001 treatment not only led to a decrease in cancer cell proliferation via apoptosis induction but also had synergistic effects with irradiation and showed anti-migratory potential in both tested cell lines (Cal27 and SCC154). The antineoplastic potential seems to be higher in SCC154, supporting the hypothesis of HPV-induced WBC pathway deregulation. Indeed, a strong downregulation of WBC transcriptional targets was observed in the HPV-positive cell line SCC154 treated with ICG-001. Furthermore, high CBP expression could possibly facilitate the identification of low-risk patients with HPV-positive HNSCC. Finally, ICG-001 may become a viable therapy option for these low-risk patients, particularly as a component of proposed treatment de-escalation regimens for this patient group. Indeed, further research is warranted in order to decipher the exact molecular effects of ICG-001, as well as to assess further in vitro and in vivo antineoplastic effects of ICG-001, particularly for HPV-positive HNSCC.

## Figures and Tables

**Table 1:** TCGA cohort patient characteristics and distribution into the two groups post-stratification based on *Crebbp* expression. *Crebbp*; Creb-binding protein, T; T-stage, N; N-stage, M; M-stage, Tx/Nx/Mx; unknown stage.

	*Crebbp* Low	*Crebbp* High	Total
Number of patients	17	24	41
Age, median (years)	59.5	53.5	57.0
Range (years)	41.1–68.3	40.6–71.5	40.6–71.5
T4, n	4	4	8
T3, n	3	2	5
T2, n	7	14	21
T1, n	3	3	6
Tx, n	0	1	1
N3, n	0	1	1
N2, n	13	13	26
N1, n	1	4	5
N0, n	2	6	8
Nx, n	1	0	1
M0, n	15	23	38
Mx, n	2	1	3

**Table 2:** Patient and tumor characteristics of the in-house cohort. PORT; post-operative radiotherapy. y; years, T; T-stage, N; N-stage, M; M-stage.

Patients	29		
Age, median (y)	63.7		
Range (y)	37.0–80.5		
	*n*	*%*	
Female	13	44.8	
Male	16	55.2	
T4	4	13.8	
T3	1	3.4	
T2	15	51.7	
T1	9	31.0	
N3	1	3.4	
N2	13	44.8	
N1	10	34.5	
N0	5	17.2	
M0	29	100.0	
PORT			
Yes	21	72.4	
No	8	27.6	

## Data Availability

The data that support the findings of this study are available on request from the corresponding author.

## References

[1] Mirza A.H., Thomas G., Ottensmeier C.H., King E.V. (2019). Importance of the immune system in head and neck cancer. Head Neck.

[2] Brkic F.F., Kadletz-Wanke L., Kenner L., Füreder T., Jank B., Brunner M., Heiduschka G. (2021). An analysis of distant metastasis cases from HPV-associated oropharyngeal squamous cell carcinoma. J. Craniomaxillofac. Surg..

[3] Ward M.J., Thirdborough S.M., Mellows T., Riley C., Harris S., Suchak K., Webb A., Hampton C., Patel N.N., Randall C.J. (2014). Tumour-infiltrating lymphocytes predict for outcome in HPV-positive oropharyngeal cancer. Br. J. Cancer.

[4] Timbang M.R., Sim M.W., Bewley A.F., Farwell D.G., Mantravadi A., Moore M.G. (2019). HPV-related oropharyngeal cancer: A review on burden of the disease and opportunities for prevention and early detection. Hum. Vaccin. Immunother..

[5] Combes J.D., Franceschi S. (2014). Role of human papillomavirus in non-oropharyngeal head and neck cancers. Oral Oncol..

[6] Mehanna H., Robinson M., Hartley A., Kong A., Foran B., Fulton-Lieuw T., Dalby M., Mistry P., Sen M., O’Toole L. (2019). Radiotherapy plus cisplatin or cetuximab in low-risk human papillomavirus-positive oropharyngeal cancer (De-ESCALaTE HPV): An open-label randomised controlled phase 3 trial. Lancet.

[7] Mirghani H., Blanchard P. (2017). Treatment de-escalation for HPV-driven oropharyngeal cancer: Where do we stand?. Clin. Transl. Radiat. Oncol..

[8] Iyer N.G., Dogan S., Palmer F., Rahmati R., Nixon I.J., Lee N., Patel S.G., Shah J.P., Ganly I. (2015). Detailed Analysis of Clinicopathologic Factors Demonstrate Distinct Difference in Outcome and Prognostic Factors Between Surgically Treated HPV-Positive and Negative Oropharyngeal Cancer. Ann. Surg. Oncol..

[9] Brkic F.F., Mayer C., Besser G., Altorjai G., Herrmann H., Heiduschka G., Haymerle G., Kadletz-Wanke L. (2021). Potential association of the prognostic index and survival in patients with p16-positive oropharyngeal squamous cell carcinoma. Wien. Klin. Wochenschr..

[10] Lee S.H., Koo B.S., Kim J.M., Huang S., Rho Y.S., Bae W.J., Kang H.J., Kim Y.S., Moon J.H., Lim Y.C. (2014). Wnt/β-catenin signalling maintains self-renewal and tumourigenicity of head and neck squamous cell carcinoma stem-like cells by activating Oct4. J. Pathol..

[11] Zhan T., Rindtorff N., Boutros M. (2017). Wnt signaling in cancer. Oncogene.

[12] Moon J.H., Lee S.H., Lim Y.C. (2021). Wnt/β-catenin/Slug pathway contributes to tumor invasion and lymph node metastasis in head and neck squamous cell carcinoma. Clin. Exp. Metastasis.

[13] Bello J.O., Nieva L.O., Paredes A.C., Gonzalez A.M., Zavaleta L.R., Lizano M. (2015). Regulation of the Wnt/β-Catenin Signaling Pathway by Human Papillomavirus E6 and E7 Oncoproteins. Viruses.

[14] Manzo-Merino J., Contreras-Paredes A., Vázquez-Ulloa E., Rocha-Zavaleta L., Fuentes-Gonzalez A.M., Lizano M. (2014). The role of signaling pathways in cervical cancer and molecular therapeutic targets. Arch. Med. Res..

[15] Kartha V.K., Alamoud K.A., Sadykov K., Nguyen B.C., Laroche F., Feng H., Lee J., Pai S.I., Varelas X., Egloff A.M. (2018). Functional and genomic analyses reveal therapeutic potential of targeting β-catenin/CBP activity in head and neck cancer. Genome Med..

[16] National Center for Biotechnology Information PubChem Compound Summary for CID 11238147. https://pubchem.ncbi.nlm.nih.gov/compound/icg-001.

[17] Lin Z., Li Q., Zhao Y., Lin Z., Cheng N., Zhang D., Liu G., Lin J., Zhang H., Lin D. (2021). Combination of Auranofin and ICG-001 Suppress the Proliferation and Metastasis of Colon Cancer. Front. Oncol..

[18] Chan L.S., Man O.Y., Kwok H.H., Chen L., Chan K.C., Lung H.L., Ngan R.K., Wong R.N., Lo K.W., Lee A.W. (2019). The Wnt modulator ICG 001 mediates the inhibition of nasopharyngeal carcinoma cell migration in vitro via the miR 150/CD44 axis. Int. J. Oncol..

[19] Liu Y., Chen H., Zheng P., Zheng Y., Luo Q., Xie G., Ma Y., Shen L. (2017). ICG-001 suppresses growth of gastric cancer cells and reduces chemoresistance of cancer stem cell-like population. J. Exp. Clin. Cancer Res..

[20] Arensman M.D., Telesca D., Lay A.R., Kershaw K.M., Wu N., Donahue T.R., Dawson D.W. (2014). The CREB-binding protein inhibitor ICG-001 suppresses pancreatic cancer growth. Mol. Cancer Ther..

[21] Evangelisti C., Chiarini F., Cappellini A., Paganelli F., Fini M., Santi S., Martelli A.M., Neri L.M., Evangelisti C. (2020). Targeting Wnt/β-catenin and PI3K/Akt/mTOR pathways in T-cell acute lymphoblastic leukemia. J. Cell Physiol..

[22] Rühlmann F., Windhof-Jaidhauser I.M., Menze C., Beißbarth T., Bohnenberger H., Ghadimi M., Dango S. (2019). The prognostic capacities of CBP and p300 in locally advanced rectal cancer. World J. Surg. Oncol..

[23] Hashida Y., Higuchi T., Matsumoto S., Iguchi M., Murakami I., Hyodo M., Daibata M. (2021). Prognostic significance of human papillomavirus 16 viral load level in patients with oropharyngeal cancer. Cancer Sci..

[24] Rampias T., Boutati E., Pectasides E., Sasaki C., Kountourakis P., Weinberger P., Psyrri A. (2010). Activation of Wnt signaling pathway by human papillomavirus E6 and E7 oncogenes in HPV16-positive oropharyngeal squamous carcinoma cells. Mol. Cancer Res..

[25] Muñoz-Bello J.O., Olmedo-Nieva L., Castro-Muñoz L.J., Manzo-Merino J., Contreras-Paredes A., González-Espinosa C., López-Saavedra A., Lizano M. (2018). HPV-18 E6 Oncoprotein and Its Spliced Isoform E6*I Regulate the Wnt/β-Catenin Cell Signaling Pathway through the TCF-4 Transcriptional Factor. Int. J. Mol. Sci..

[26] Lacau St Guily J., Rousseau A., Baujat B., Périé S., Schultz P., Barry B., Dufour X., Malard O., Pretet J.L., Clavel C. (2017). Oropharyngeal cancer prognosis by tumour HPV status in France: The multicentric Papillophar study. Oral Oncol..

[27] Huang Y., Sheng H., Xiao Y., Hu W., Zhang Z., Chen Y., Zhu Z., Wu D., Cao C., Sun J. (2021). Wnt/β-catenin inhibitor ICG-001 enhances the antitumor efficacy of radiotherapy by increasing radiation-induced DNA damage and improving tumor immune microenvironment in hepatocellular carcinoma. Radiother. Oncol..

[28] Gökyildirim M.Y., Grandel U., Hattar K., Dahlem G., Schuetz E., Leinberger F.H., Eberle F., Sibelius U., Grimminger F., Seeger W. (2018). Targeting CREB-binding protein overrides LPS induced radioresistance in non-small cell lung cancer cell lines. Oncotarget.

[29] Schultz J.D., Sommer J.U., Hoedt S., Erben P., Hofheinz R.D., Faber A., Thorn C., Hörmann K., Sauter A. (2012). Chemotherapeutic alteration of β-catenin and c-kit expression by imatinib in p16-positive squamous cell carcinoma compared to HPV-negative HNSCC cells in vitro. Oncol. Rep..

[30] Lorusso P.M. (2016). Inhibition of the PI3K/AKT/mTOR Pathway in Solid Tumors. J. Clin. Oncol..

[31] Wang L., Wang W. (2021). Safety and efficacy of anaplastic lymphoma kinase tyrosine kinase inhibitors in non-small cell lung cancer (Review). Oncol. Rep..

[32] Kimura K., Ikoma A., Shibakawa M., Shimoda S., Harada K., Saio M., Imamura J., Osawa Y., Kimura M., Nishikawa K. (2017). Safety, Tolerability, and Preliminary Efficacy of the Anti-Fibrotic Small Molecule PRI-724, a CBP/β-Catenin Inhibitor, in Patients with Hepatitis C Virus-related Cirrhosis: A Single-Center, Open-Label, Dose Escalation Phase 1 Trial. EBioMedicine.

[33] Lenz H.J., Kahn M. (2014). Safely targeting cancer stem cells via selective catenin coactivator antagonism. Cancer Sci..

[34] El-Khoueiry A.B., Ning Y., Yang D., Cole S., Kahn M., Zoghbi M., Berg J., Fujimori M., Inada T., Kouji H. (2013). A phase I first-in-human study of PRI-724 in patients (pts) with advanced solid tumors. J. Clin. Oncol..

[35] Ko A.H., Chiorean E.G., Kwak E.L., Lenz H.J., Nadler P.I., Wood D.L., Fujimori M., Inada T., Kouji H., McWilliams R.R. (2016). Final results of a phase Ib dose-escalation study of PRI-724, a CBP/beta-catenin modulator, plus gemcitabine (GEM) in patients with advanced pancreatic adenocarcinoma (APC) as second-line therapy after FOLFIRINOX or FOLFOX. J. Clin. Oncol..

[36] Deng J., Hua L., Han T., Tian M., Wang D., Tang H., Sun S., Chen H., Cheng H., Zhang T. (2020). The CREB-binding protein inhibitor ICG-001: A promising therapeutic strategy in sporadic meningioma with NF2 mutations. Neurooncol. Adv..

[37] Mirghani H., Amen F., Blanchard P., Moreau F., Guigay J., Hartl D.M., Lacau St Guily J. (2015). Treatment de-escalation in HPV-positive oropharyngeal carcinoma: Ongoing trials, critical issues and perspectives. Int. J. Cancer.

[38] The Cancer Genome Atlas Program. https://www.cancer.gov/tcga.

[39] Schneider C.A., Rasband W.S., Eliceiri K.W. (2012). NIH Image to ImageJ: 25 years of image analysis. Nat. Methods.

